# Stroke Mimics in a Tertiary Comprehensive Stroke Center: Implications for Healthcare Provider Education

**DOI:** 10.7759/cureus.116916

**Published:** 2026-09-25

**Authors:** Mohammad Hossein Abbasi, Andrew Helson, Angel Penafiel, Alice S Wang, Dan E Miulli, Farrukh S Khan

**Affiliations:** 1 Neurology, Arrowhead Regional Medical Center, Colton, USA; 2 Neurosurgery, Riverside University Health System Medical Center, Moreno Valley, USA; 3 Neurosurgery, Riverside University Health System, Moreno Valley, USA; 4 Neurosurgery, Arrowhead Regional Medical Center, Colton, USA

**Keywords:** acute ischemic stroke (ais), code stroke, quality improvement initiative, stroke mimics, stroke triage

## Abstract

Background: The proportion of stroke mimics in code stroke activation varies. Decreasing the number of stroke mimics requires clinicians to master the skills needed to distinguish strokes from stroke mimics. There is a lack of quality initiatives to produce such astute clinicians, who, once trained, should improve the accuracy of stroke alerts, reduce patient risk from unnecessary radiation, optimize resource utilization, and correctly diagnose patients to provide the best treatment course.

Methodology: As a comprehensive stroke program, stroke data were collected as part of the continuous quality control and improvement procedures. A retrospective study from January 2026 to June 2026 was conducted using the original stroke database to select patients with code stroke activation who were treated with the code stroke protocol and their final diagnoses at discharge. No individual patient data were reviewed. No inferential statistical analyses were performed.

Results: From January 2026 to June 2026, there were a total of 630 code stroke activations (mean = 105 patients/month; range = 86-120 patients). True strokes accounted for 35.1% (221/630, 35.1%) of the code stroke activations, yielding 64.9% (409/630, 64.9%) stroke mimics. Among stroke patients, 110/630 (17.5%) were ischemic strokes, 96/630 (15.2%) were transient ischemic attacks, 14/630 (2.2%) were intracerebral hemorrhages, and 1/630 (0.2%) was a subarachnoid hemorrhage. The stroke mimics were sepsis (36/630, 5.7%), migraine (29/630, 4.6%), seizure (21/630, 3.3%), subdural hematoma (21/630, 3.3%), hypertensive emergency/crisis (21/630, 3.3%), residual deficits from prior stroke (18/630, 2.9%), unruptured aneurysm (13/630, 2.1%), alcohol abuse/other drug use/mental illness (12/630, 1.9%), Bell’s palsy (9/630, 1.4%), and all other diagnoses (229/630, 36.3%).

Conclusions: In this study, stroke mimics accounted for 409/630 (64.9%) of code stroke activations, which is higher than documented in the literature and in past performance at this institution. The most likely stroke mimics are sepsis, migraine, seizure, subdural hematoma, and hypertensive emergency/crisis. Parallel increases in public awareness and education on stroke and lifelong education on acute stroke recognition must continue for all clinicians to accurately identify patients with acute strokes, for which a large amount of hospital resources is used for code stroke activation. Future studies should investigate methods to efficiently diagnose patients and optimize healthcare resources.

## Introduction

Stroke mimics are encountered in the daily care of patients with acute stroke alert activation. Therefore, screening tools such as the Cincinnati Prehospital Stroke Scale (CPSS) and the modified Los Angeles Prehospital Stroke Screen (mLAPSS) are utilized to improve the accuracy of code stroke activation and to optimize resource utilization in comprehensive stroke centers [1-3]. These tools screen for balance loss, eye changes, face drooping, arm weakness, and speech difficulty [2]. The modified Los Angeles Prehospital Stroke Screen (mLAPSS), used by paramedics taking patients to this institution, also records exclusions due to a history of seizure or epilepsy, patients younger than 40 years of age, patients who are wheelchair-bound or bedridden, blood glucose less than 60 mg/dL or over 400 mg/dL, and no weakness or bilateral weakness [2]. The mLAPSS is 91% sensitive and 97% specific for quickly identifying potential stroke patients in the emergency setting [3]. Despite the widespread use of prehospital stroke screening tools, previous studies have found that approximately 5% to 50% of patients who had code stroke activation were stroke mimics, not strokes [4-9]. The increased number of code stroke activations for stroke mimics using standard protocols may be attributed to increased public education to call 9-1-1 for stroke-like symptoms. More public awareness of stroke-like symptoms is advantageous in reducing the chance of missing patients with strokes; on the other hand, people presenting to the emergency department may be subjected to unnecessary imaging radiation, treatment interventions, hospital visits, or hospital stays [10-13]. Adhering to the culture that “time is brain,” modern treatment algorithms assess potential stroke patients in the ambulance bay, determine hemodynamic stability, confirm stroke history, symptoms, and signs from the patient, family, or emergency management personnel, perform a neurological exam, obtain blood glucose, and send patients for imaging studies, all within the first five minutes of patient arrival. A full acute stroke workup may include computed tomography (CT) of the head, CT angiography, CT perfusion studies, and/or other imaging. Along with imaging findings, clinical presentation and the National Institutes of Health Stroke Scale (NIHSS), performed upon presentation, may indicate some patients for thrombolytic therapy [7]. A recent report found that among patients who received intravenous thrombolysis, 29% of patients who had low NIHSS scores and disabling symptoms actually had stroke mimics, not strokes [7]. Here, we describe the recent and recurrent trend in the presentation of increased stroke mimics at this tertiary comprehensive stroke center. The findings from this current study may help guide future quality initiatives to improve the accuracy of stroke alerts, increase continuous stroke education, and optimize resource utilization.

## Materials and methods

This is an Institutional Review Board (IRB)-approved retrospective study (Protocol #20-26) at Arrowhead Regional Medical Center in Colton, California, to investigate neurosurgical comorbidities and outcomes. Data from hospital-based metric records for stroke care are routinely collected for quality improvement, stroke center certification by the stroke program, and identification of trends to improve performance. Therefore, these data, specifically from January 2026 to June 2026, were used in this study; no individual patient charts were reviewed, and no individual patient data were collected. The inclusion criteria included all patients >18 years old who had code stroke activation and were treated with the code stroke protocol. All other patients who were <18 years old and did not have code stroke activation were excluded from this study. For each code stroke activation, a final ICD-10 code (International Classification of Diseases, 10th Revision) was assigned, and this was used to identify patients with strokes and those without strokes or with stroke mimics. The data were then further split into strokes and stroke mimics to investigate stroke type and specific stroke mimics. Strokes were defined as transient ischemic attack, ischemic stroke, intracerebral hemorrhage, or subarachnoid hemorrhage. Other diagnoses were considered stroke mimics. Descriptive statistics were used, and no inferential analyses were conducted.

## Results

There were a total of 630 code stroke activations (mean = 105 patients/month; range = 86-120 patients) during the six months examined (Table 1). True strokes were found in (221/630, 35.1%) of the original stroke activations, and (409/630, 64.9%) were stroke mimics. In this study, (110/630, 17.5%) were ischemic strokes, (96/630, 15.2%) were transient ischemic attacks, (14/630, 2.2%) were intracerebral hemorrhages, and (1/630, 0.2%) was a subarachnoid hemorrhage. The stroke mimics were sepsis (36/630, 5.7%), migraine (29/630, 4.6%), seizure (21/630, 3.3%), subdural hematoma (21/630, 3.3%), hypertensive emergency/crisis (21/630, 3.3%), residual deficits from prior stroke (18/630, 2.9%), unruptured aneurysm (13/630, 2.1%), alcohol abuse/other drug use/mental illness (12/630, 1.9%), Bell’s palsy (9/630, 1.4%), and all other diagnoses (229/630, 36.3%) (Figure 1). The *all other diagnoses* category included all other ICD-10 codes, many of which were counted only once or twice. The red box in Figure 1 included all the stroke mimics.

**Table 1:** Total number of code stroke activations, mean number of code stroke activations per month, and range from January 2026 to June 2026.

January 2026 to June 2026	Number of patients
Code stroke activation	630
Mean code stroke activations per month	105
Range	86-120

**Figure 1:**
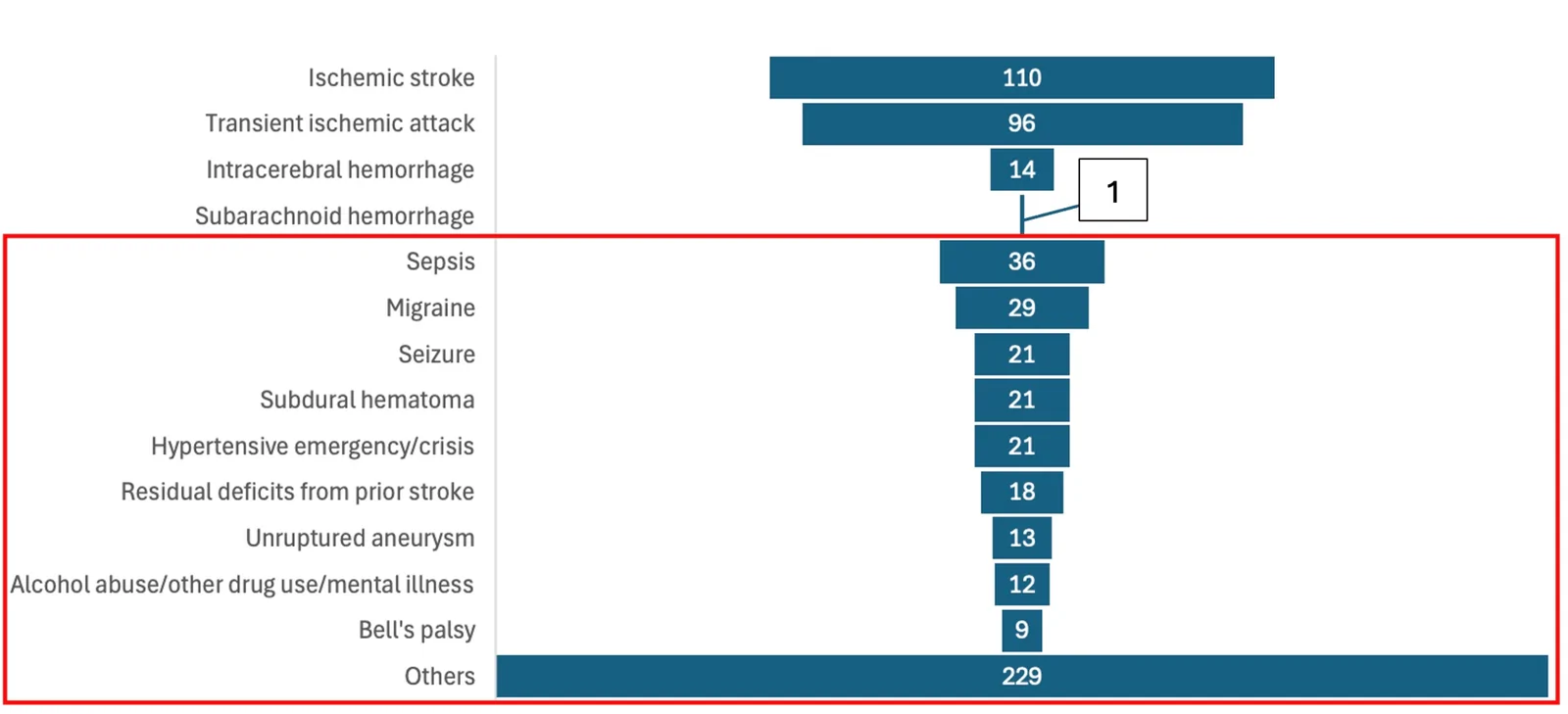
Diagnoses at discharge of all 630 code stroke activations. Strokes include ischemic strokes, transient ischemic attacks, intracerebral hemorrhage, and subarachnoid hemorrhage. The red box shows the most common diagnoses that accounted for stroke mimics in our study. The total number of patients with each diagnosis is shown.

## Discussion

A high stroke mimic rate alone does not necessarily indicate inappropriate stroke activation, as highly sensitive stroke systems may intentionally accept lower specificity to minimize missed strokes. In this study, 409/630 (64.9%) of all code stroke activations within a period of six months were stroke mimics. These stroke mimics have dramatically increased at this institution from previous years. Clinical presentations vary among different stroke patients, who can have either ischemic stroke, transient ischemic attack, intracerebral hemorrhage, or subarachnoid hemorrhage. Subdural hematoma is not considered a stroke. Distinguishing stroke types solely based on clinical presentation should not be so challenging for a comprehensive stroke center. Pathologies mimicking strokes are known as stroke mimics. Previous studies have found a wide range of stroke mimics, from 5% to 50% [4-9]. This study demonstrates a higher percentage of stroke mimics (64.9%) than that reported in the literature, in addition to that in the multiple years before this study. The discrepancies may be multifactorial and can depend on the level of public awareness regarding stroke-like symptoms, location and size of a stroke center (county versus city), familiarity of clinicians working with stroke and non-stroke patients, ability and accessibility to obtain care for healthcare problems, social determinants of health, and others. The percentage of hemorrhagic strokes decreased dramatically at this institution from a high of 40% to the current 11%, which can lead to more reliance on the nuances of the neurological exam than before to accurately diagnose stroke.

In this cohort, the observed proportion of stroke mimics is higher than previously reported but similar to the historic data from the year before the institution’s application to become a comprehensive stroke center [4-9]. During that application process, multiple measures were put into place. The increased number of stroke mimics entering the code stroke protocol may reflect the increased number of patients presenting with neurological symptoms or signs, over 1,800 per year, overloading the emergency department, as well as more public education on recognizing stroke-like symptoms, increased public awareness of stroke-like symptoms, and heightened vigilance among emergency department staff and inpatient staff to triage potential stroke presentations [14]. Thus, the evolution of code stroke activation has led to a high volume of patients entering the acute stroke protocol while more underinsured patients present to the county hospital emergency department with advanced diseases previously treated by primary care providers. Since more underinsured patients are not visiting primary care physicians, these patients might not be familiar with their health history, with the exception of their current symptomatology. These stroke-like patients undergoing acute diagnosis and treatment need to have stroke care in the shortest amount of time. This care includes imaging with radiation exposure. For example, patients with stroke mimics also undergo CT imaging, which may include CT head, CT angiography, and CT perfusion, with each exposure adding additional radiation [10-12]. Because stroke mimics tend to occur in younger patients, the cumulative radiation risk may be greater given their longer remaining life expectancy [12]. Once the initial assessment of a clinical vascular localization supports acute stroke onset, a CT head and CT angiography determination of a physiological vascular abnormality would help separate the several differential diagnoses of sepsis, migraine, Todd’s paralysis, functional deficit, intoxication, and Bell’s palsy. It should be noted that an arterial stenosis or chronic occlusion can be incidental, so anatomical concordance is still required.

This study also reviewed the type of stroke education provided to the departments of nursing, emergency medicine, neurology, and neurointervention personnel, as well as to the new residents of neurology and radiology at the institution. We observed a correlation. Stroke education became less dependent upon neurosurgery physicians during this time. The type of lecture also changed from an emergent assessment of stroke that included the best-performing signals of prehospital screening reports, medical history, acute stroke abrupt onset, focal or lateralizable deficit, vascular-territory coherence, and negative symptoms to the role of thrombectomy. The number of stroke lectures also dropped from 10 to 5, participation in didactic-based education decreased from 95% to 80%, mock code stroke and code stroke large vessel occlusion scenarios went from 6 to 0, and code stroke algorithm displays in the emergency department went from 24 to 0, demonstrating a relative lack of education in the 12 months prior to the current review. Education in stroke diagnosis and treatment is paramount. An increase in hands-on stroke education for all providers, mock code strokes, frequency of education with scenarios, and altering the current code stroke protocol for the current patient population, which was previously conducted but fell away, would once again be beneficial, specifically if all can be performed on a monthly basis.

Yet, it is not beneficial to reduce the number of actual code stroke activations that would benefit from acute stroke care or the number of imaging studies that would help rule out certain diagnoses such as subdural hematoma. In an acute setting with limited information and time, an astute clinician must be able to distinguish between strokes and stroke mimics. This has been challenging because of overlapping clinical presentations between strokes and stroke mimics and when labs and imaging studies are not yet available to help determine the diagnosis [8]. As demonstrated in this study, most of the stroke mimics were sepsis, migraine, seizure, subdural hematoma, hypertensive emergency or crisis, residual deficits from prior stroke, unruptured aneurysm, and alcohol abuse/other drug use/mental illness, as seen in the red box in Figure 1. The role of an astute clinician is to master the ability to observe subtle neurological details in similar clinical presentations, assemble pieces of information into a coherent, logical picture, and determine the need for certain sets of labs and imaging studies to support the most likely diagnosis. To reduce unnecessary workup and optimize resource utilization, we recommend some specifics to observe. Overall, an astute clinician must note the report from prehospital stroke screening, medical history, nature of onset, positive or negative symptoms, anatomical localization to neurological deficits, level of consciousness, known deficits, and vital signs. In general, abrupt onset with focal neurological deficits in a vascular territory, without a significant medical history, favors strokes, while gradual evolution with multi-organ involvement favors stroke mimics. An acute onset of symptoms, transient or permanent, that can be localized to a specific vascular territory, small or big, would favor strokes. For example, abnormal temperature, hemodynamic instability, and metabolic derangements would favor sepsis; progressively worsening visual/sensory phenomena would favor migraine; gaze deviation with tongue biting, rhythmic activity, and confusion afterward would favor seizure; history of anticoagulation/head trauma and cognitive decline with progressively worsening focal neurological deficits would favor subdural hematoma; history of hypertension with headache, confusion, and visual changes would favor hypertensive emergency/crisis; history of strokes with stable residual deficits would favor old stroke, not a new diagnosis of stroke; slow, progressively worsening cranial neuropathy would favor a growing unruptured aneurysm; history of drug use with global encephalopathy would favor drug abuse; and history of mental illness, poor historian, and inconsistency in exams would favor functional or psychiatric disorder. Once the most likely diagnosis is made from clinical observations, certain labs or imaging studies to order should become obvious or logical. We believe the most important skill of an astute clinician is to localize the lesion to a vascular territory based on clinical presentations and neurological exam.

Another objective modality other than history, vitals, neurological exam, and correlation to a vascular territory that can potentially be used in the acute, time-sensitive setting is magnetoencephalography (MEG). Increased delta magnetic activity correlated with NIHSS was found in acute ischemic stroke patients (*n* = 18 patients) [15]. More interestingly, in a small cohort of eight patients with transient ischemic attacks (TIAs) or transient global amnesia (TGAs), Stippich et al. found higher focal slow activity (2-6 Hz) up to 14 times the normal values from sensorimotor and mesiotemporal cortices during the first hours after the attack, which normalized in eleven days [16]. Brazdzionis et al. compared neural electromagnetic fields measured from healthy subjects and stroke patients. In the acute setting, stroke patients had highly variable but more dramatic morphologic changes of injury within the 1-3 Hz range and fewer variations in the waveforms above 4 Hz compared to the healthy subjects [17]. These studies highlight the potential to use a portable MEG helmet in acute settings to determine stroke patients. However, larger studies are needed to investigate the validity of this study and the sensitivity and specificity of specific MEG findings to certain pathologies.

Limitations 

This is a single institution study with a short time frame of six months, which may limit its generalizability. Future studies should include large patient populations with longer study time frames to evaluate the validity of this study's findings. This study also relies on the accuracy of coding the correct diagnosis at discharge. It is possible that there may be human error in entering the correct ICD-10 codes. These ICD-10 codes were not independently adjudicated through individual chart review, creating potential diagnostic misclassification. Continual education on how to select the most representative ICD-10 code can help with accurate coding. Another aspect of this study is that it only includes patients with code stroke activation and does not capture patients who screened negative or strokes that may have been missed; therefore, sensitivity, specificity, and overall diagnostic accuracy of the screening process cannot be determined. Finally, healthcare provider education was not formally evaluated as an exposure, precluding causal conclusions regarding its relationship with stroke mimic rates. Therefore, future studies should investigate whether education decreases the number of stroke mimics.

## Conclusions

The ability of the public to recognize stroke-like symptoms has evolved, likely due to improved public education and awareness, to the point that there is an increasing trend of stroke mimics in code stroke activation. There has also been an increase in underinsured patients and an increase in emergency room visits at county hospitals. While it is advantageous to recognize patients with strokes, it is disadvantageous for patients who are stroke mimics to undergo the entire stroke protocol. Strokes are specific diagnoses made by multiple healthcare providers in an acute care setting. As an increasing number of stroke-like patients present to the emergency department, in our case over 1,800 per year, with a concurrent increase in patients presenting for first-time healthcare to the county hospital with more advanced diseases, it may seem prudent to return to advanced education goals. We believe the goal is to produce astute clinicians who can localize the stroke lesion based on clinical presentations, which is to say, the prehospital stroke screening report, the nature of onset of symptoms, history of medical conditions, positive or negative symptoms, anatomical localization to neurological deficits, level of consciousness, known deficits, and vitals before lab and imaging workup. Future studies should focus on developing a standardized protocol to educate healthcare providers to accurately identify patients with acute strokes and optimize utilization of healthcare resources.
